# Retinal Sensitivity in Comparison to Cone Density in Choroideremia

**DOI:** 10.1167/iovs.65.14.6

**Published:** 2024-12-03

**Authors:** Niamh Wynne, Yu You Jiang, Tomas S. Aleman, Jessica I. W. Morgan

**Affiliations:** 1 Scheie Eye Institute, University of Pennsylvania, Perelman School of Medicine, Philadelphia, Pennsylvania, United States; 2 Center for Advanced Retinal and Ocular Therapeutics, University of Pennsylvania, Philadelphia, Pennsylvania, United States

**Keywords:** adaptive optics imaging, choroideremia, retinal sensitivity

## Abstract

**Purpose:**

Choroideremia (CHM) is an X-linked inherited retinal degeneration causing loss of photoreceptors, retinal pigment epithelium, and choriocapillaris. Structural abnormalities of the cone photoreceptor mosaic have been reported even within the retained island of functioning retina. Here, we describe the relationship between cone density and visual sensitivity within the retained central retina in CHM.

**Methods:**

The cone mosaics of 31 patients aged 10 to 57 years with CHM were imaged using a multi-modal, custom-built adaptive optics scanning light ophthalmoscope (AOSLO). Retinal sensitivity was measured using fundus tracking perimetry, cross-sectional retinal structure with optical coherence tomography. The relationship between sensitivity at each retinal location tested and structural parameters of local disease severity (cone density and distance to border) was explored using unpaired *t*-tests and linear regressions. Ellipsoid zone area was also investigated.

**Results:**

Three hundred fourteen individual regions of interest were analyzed. Cone density and retinal sensitivities were significantly decreased compared with healthy controls (*P* < 0.0002). There was a statistically significant correlation between cone density and visual sensitivity within the fovea and parafovea (*P* < 0.0005), but not in the perifovea (*P* > 0.1). There was a significant relationship between the distance to the atrophic border and sensitivity from 400 µm to 1600 µm eccentricity (*P* < 0.001). Mean sensitivity was significantly related to the ellipsoid extent (*P* < 0.0001).

**Conclusions:**

The correlation between cone density and retinal sensitivity within 1 mm of the foveal center in CHM suggests central sensitivity loss is driven by cone loss. Outside of the central fixation point, proximity to the atrophic border is correlated with sensitivity, suggesting the presence of a functional transition zone.

Choroideremia (CHM) is an X-linked inherited retinal degeneration that affects approximately 1 in 50,000 individuals. The disease is caused by pathogenic variants in the *CHM* gene which codes for Rab-escort protein 1 (REP-1). REP-1 is a ubiquitously expressed protein involved in intracellular vesicular trafficking, including melanosomes.^1^ Patients with CHM present with nyctalopia and difficulties recognizing and avoiding obstacles caused by mid-peripheral scotomas, corresponding to progressive atrophy of photoreceptors, retinal pigment epithelium (RPE), and choroid. The clinical course involves progressive visual field loss and legal blindness by the fourth decade of life, although central vision can be maintained late into the disease course.^2^ Abnormal macular function is known to occur in CHM even in individuals with normal or relatively spared visual acuity, although the precise relationship between vision loss and the underlying local cellular changes remain less explored.^3^^,^^4^

Adaptive optics scanning light ophthalmoscopy (AOSLO) is an advanced imaging technique capable of resolving single cells in the living retina, therefore providing the opportunity to monitor structural disease progression on a cellular scale.^5^ AOSLO studies of the photoreceptor mosaic in CHM have revealed qualitative and quantitative abnormalities in cone photoreceptor mosaic even within the relatively spared island of the functioning retina,^6^^–^^10^ and a sharp decline in visual sensitivity at the atrophic border.^11^ Although monitoring the photoreceptor mosaic using AOSLO offers an opportunity to structurally surveil the retina, an understanding of the structure function relationship in CHM is necessary to optimize clinical trial design and facilitate appropriate end point selection. The purpose of this study was to examine the extent of the retained island of the functioning retina, assess the structural health of the cone photoreceptor mosaic in comparison with visual sensitivities, and improve our understanding of this structure-function relationship in CHM.

## Methods

This study was approved by the Institutional Review Board at the University of Pennsylvania and adhered to the tenets of the Declaration of Helsinki for research involving human participants. Each participant gave informed consent (and parental consent and assent for those under 18 years of age) prior to enrollment in the study.

### Participants

Thirty-one patients with a clinical diagnosis of CHM were recruited for the present study. Thirty patients had molecularly confirmed disease, whereas 1 patient had a normal *CHM* gene sequence, suggesting a possible noncoding variant. The average age of the participants at the time of imaging was 29 years (range = 10–57 years) and all patients had participated in previous studies from our broader group.^7^^,^^9^^–^^14^ Axial length was measured using an IOL Master (Carl Zeiss Meditec USA, Dublin, CA, USA).

### Visual Function Testing

Visual acuity was measured using the Early Treatment Diabetic Retinopathy Study (ETDRS) visual acuity chart. Fundus tracking microperimetry (Nidek MP-1; CenterVue Inc., Fremont, CA, USA) was used to measure retinal sensitivity to a Goldman size III white stimulus presented for 200 ms on a background of 1.27 cd/m^2^ at locations every 1.5 degrees from the fovea to 12 degrees along meridians at 0 degrees, 90 degrees, 180 degrees, and 270 degrees. A total of 41 locations were tested for each individual. A white circle of 0.5 degrees diameter was used as a fixation target with “fade-out” protection on. A staircase 4-2 thresholding algorithm was used. Sensitivity measurements were recorded on a scale of 0 decibels (dB) to 20 dB. Mean sensitivities used were taken directly from the Nidek MP-1 device and manually calculated, excluding the areas within the established region of atrophy for each subject to minimize the floor effect of regions with no sensitivity. Regions with 0 dB sensitivity found within the residual island of the retina were included in this calculation.

### Optical Coherence Tomography Acquisition and Analysis

A spectral domain optical coherence tomography (SD-OCT) system was used for cross-sectional retinal imaging (Spectralis; Heidelberg Engineering, Heidelberg, Germany). There were 6 × 6 mm or 9 × 7 mm macular raster (or volume) scans that were acquired crossing the center of the fovea horizontally and vertically. En face projection of the ellipsoid zone (EZ) band was used to visually identify the transition from residual island to atrophic retina. The termination of the EZ band on optical coherence tomography (OCT) was taken as definitive of the atrophic border. Measurements of the retained island were then calculated by using the caliper measurement and analysis tools available within the Spectralis Heyex software (version 1.10.12.0). Measurements were scaled by a magnification factor for each subject equal to the subject's axial length in millimeters (mm) divided by 24 mm to account for axial length differences.

### AOSLO Image Acquisition and Analysis

**Figure 1.**
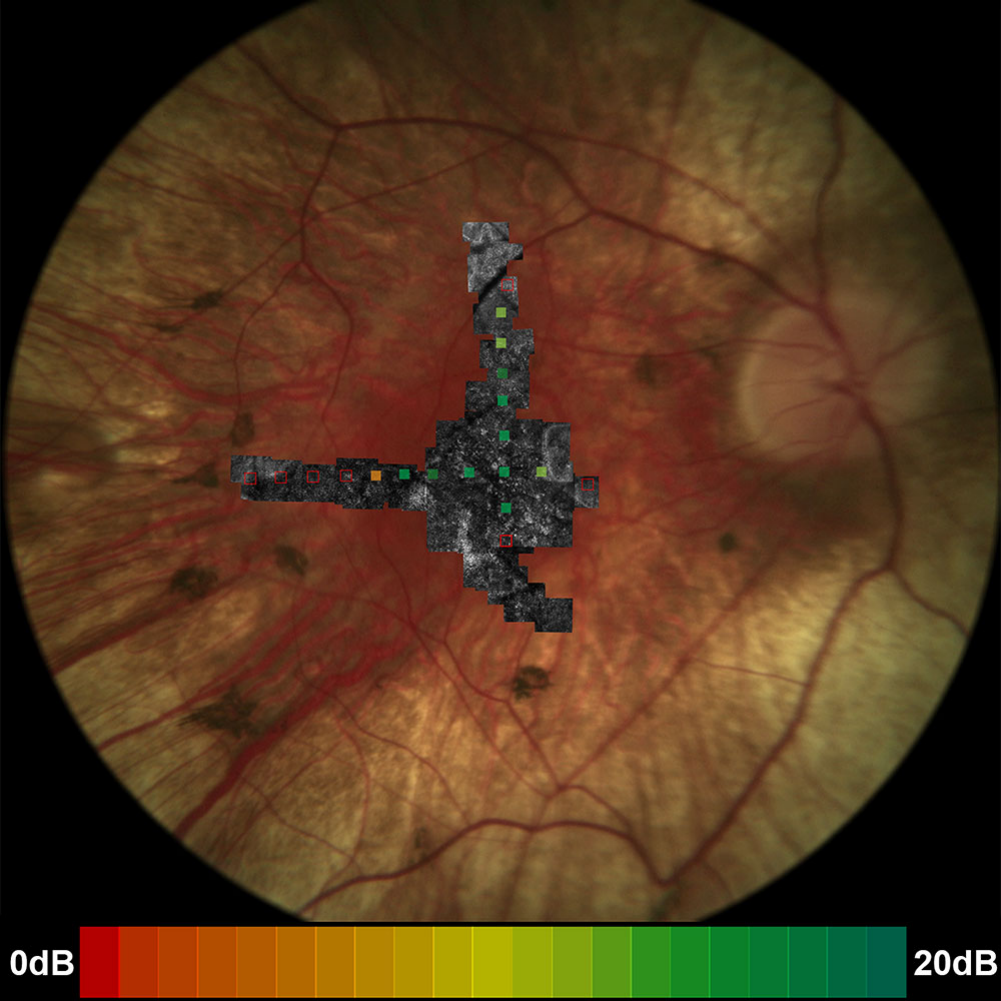
A 30 degree color fundus photograph of the right eye of a 32-year-old participant demonstrating classical pale pigmentation peripherally secondary to photoreceptor, retinal pigment epithelial, and choroidal atrophy. Superimposed is the aligned confocal AOSLO montage. Locations of 70 µm × 70 µm ROIs are indicated by *colored squares*. Color of the *filled in squares* corresponds to the visual sensitivity recorded in this region, with *empty red squares* indicating that no response was recorded to the brightest visual stimulus. The key on the bottom indicates the corresponding sensitivity values from 0 to 20 dB.

All participants were imaged using a custom-built AOSLO.^15^^,^^16^ Multi-modal images were captured for 27 participants, whereas 4 had only confocal imaging performed. Imaging protocol included a 3 × 3 degree region surrounding the fovea, and 4 strips in the superior, inferior, nasal, and temporal directions. Imaging extended to the atrophic border in each direction or to approximately 12 degrees from the fovea. Prior to imaging, the pupils were dilated using a drop of 1% tropicamide and 2.5% phenylephrine ophthalmic solutions. Bite bar stabilization was used. Images were desinusoided, registered,^17^ and automatically montaged^18^ with manual correction and sorted for quality.^19^ The eye with the highest quality AOSLO images and/or the greatest extent of imaging from each participant was selected for analysis when data from both eyes were available. Gross alignment of AOSLO images and microperimetry was achieved by aligning large structural features, such as blood vessels between the AOSLO montage and the fundus image provided with the microperimetry responses. Minor adjustments were made to align the preferred retinal locus of fixation between modalities (Fig. 1). The image scale in pixels/mm was determined using the pixels/degree of the AOSLO system, assuming 291 microns/degree for an eye with an axial length of 24 mm and multiplying by a magnification factor equal to the ratio of each subject's axial length in mm divided by 24 mm. Then, 70 µm × 70 µm regions of interest (ROIs) were cropped from the AOSLO images from regions corresponding to where microperimetry stimuli were presented. Cones were automatically identified with manual correction (Mosaic, Translational Imaging Innovations). Non-confocal split detection AOSLO images of the cone inner segments, where available, were used for disambiguation of rods and multi-modal cones. Cone coordinates were used to generate values for bound cone density. ROIs were binned by eccentricity with the following bins: 0 µm (fixation), 400 µm, 800 µm, 1200 µm, 1600 µm, and 2000 µm. The nearest atrophic border to each ROI was identified by assessing across imaging modalities and the distance to the atrophic border defined as the closest location of photoreceptor loss (either by loss of cones on AOSLO or loss of the EZ on OCT) for each ROI was measured.

### Statistical Analyses

Each participant had a single eye included in the analysis. For the purposes of statistical analysis and visualization, regions where the brightest stimulus (0 dB) was not seen were given a value of −1. Visual sensitivity measurements and cone density in CHM were compared with those previously reported in healthy controls using unpaired *t*-tests.^20^^,^^21^ The Kruskal Wallis test was used to compare average visual sensitivities and cone densities across different eccentricities. The relationships between visual sensitivity and cone density and distance to the atrophic border, respectively, at each eccentricity bin were examined using linear regression. Linear regression also was used to compare the retained EZ band area to the mean sensitivity. Statistical significance was defined as *P* < 0.05.

## Results

The ROIs (*n* = 409) were cropped from AOSLO montages that contained sensitivity estimates by microperimetry. Of these, 339 ROIs had resolvable cones across the ROI, and thus were included for further analysis. Twenty-five ROIs were excluded due to being outside of the eccentricity bins selected for analysis after the multimodal alignment of the AOSLO montage to the microperimetry pattern. Therefore, 314 ROIs were used for further analysis. All eccentricities examined had a minimum of 22 data points from a minimum of 10 participants.

**Figure 2.**
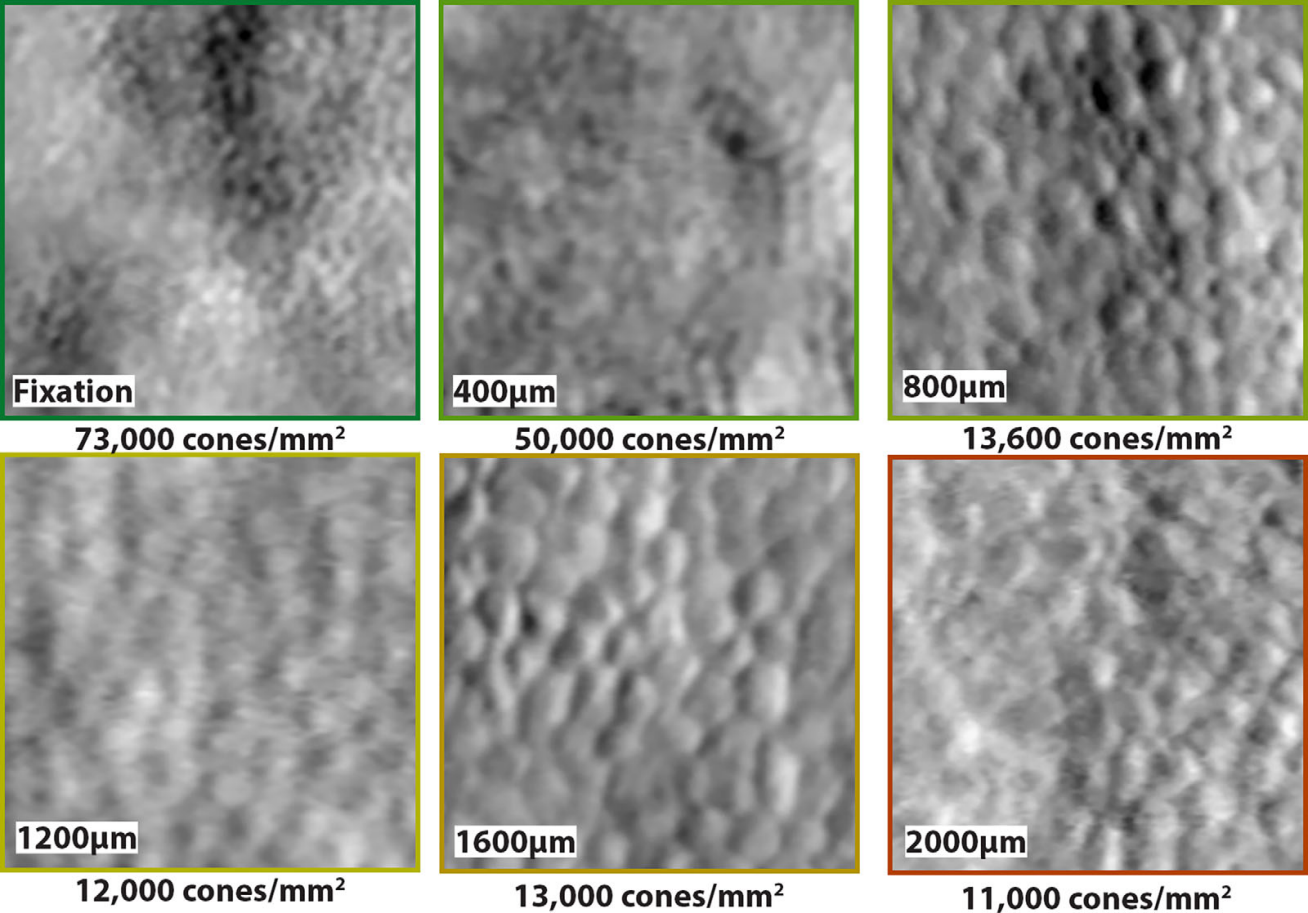
Examples of split detection AOSLO images across eccentricities. Each panel is a 70 µm × 70 µm ROI that was used to generate cone density measurements for the corresponding eccentricity. Panels were taken from different participants. Colored border of each panel corresponds to the visual sensitivity recorded at this location using the same scale as shown in Figure 1.

**Figure 3.**
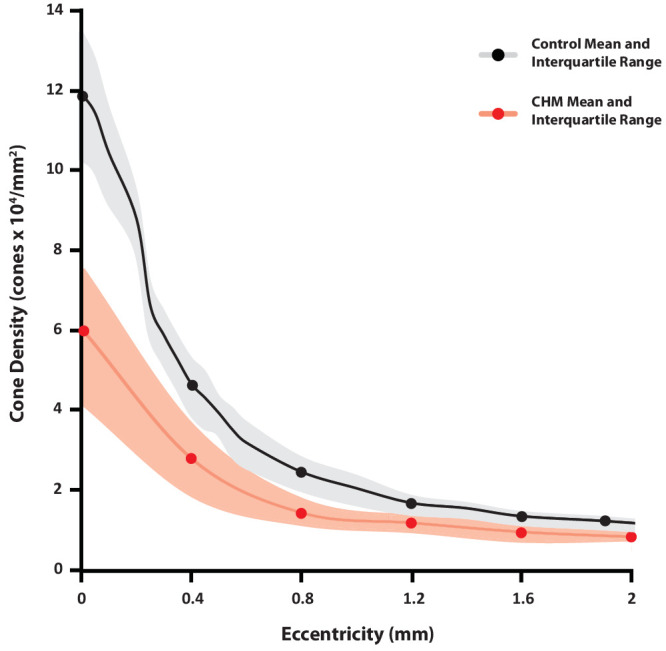
Cone density with eccentricity in CHM compared with controls. Normal-sighted control data are shown in *black*, and CHM data in *r*ed. Normal-sighted control data are taken from Cooper et al.^21^ *Shaded areas* represent the interquartile range. At the region of fixation, CHM cone density is approximately 50% reduced compared with normal-sighted controls. Beyond 1 mm, cone density in CHM is closer to normal, but still statistically significantly reduced.

Representative examples of ROIs with corresponding cone densities for each eccentricity are shown in Figure 2. Average cone density at fixation in patients with CHM was 59,800 (*n* = 25 ROIs, SD = 21,700) cones/mm^2^, at 400 µm eccentric it was 27,500 (*n* = 86, SD = 13,000) cones/mm^2^, at 800 µm was 15,100 (*n* = 87, SD = 6300) cones/mm^2^, at 1200 µm was 11,400 (*n* = 64, SD = 2800) cones/mm^2^, at 1600 µm was 9800 (*n* = 32, SD = 2800) cones/mm^2^, and at 2000 µm was 8400 cones/mm^2^ (*n* = 22, SD = 2800; Fig. 3). Cone density was significantly decreased compared with healthy controls at all locations (fixation, 400 µm, 800 µm, 1200 µm, and 2000 µm: all *P* < 0.0001; and 1600 µm, *P* = 0.0002) Average cone densities differed across eccentricities (*P* < 0.0001).

Mean visual acuity was 80 ETDRS letters (Snellen equivalent 20/25) with a range of 52 to 89 ETDRS letters (20/16-1 to 20/100+2 Snellen equivalent). Average sensitivity estimates by microperimetry were always abnormal. Over the 314 ROIs included for analysis, the average microperimetry sensitivity in CHM at fixation was 13 dB (SD = 4.8), at 400 µm was 13 dB (SD = 6.4), at 800 µm was 12 dB (SD = 6.3), at 1200 µm was 11 dB (SD = 6.4), at 1600 µm was 11 dB (SD = 6.6), and at 2000 µm was 9 dB (SD = 5.7). Microperimetry sensitivities were different on average across each eccentricity tested (*P* = 0.04). There was a statistically significant reduction in microperimetry sensitivities compared with healthy controls in all sectors (*P* < 0.0001).^20^

**Figure 4.**
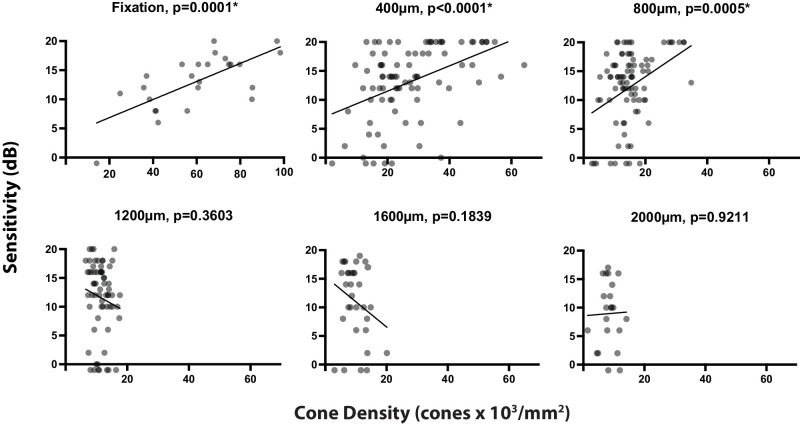
Visual sensitivity compared to cone density across eccentricities. Each panel shows the linear regression comparing microperimetry response with cone density at each respective eccentricity. Locations where no sensitivity response was recorded to the brightest visual stimulus are plotted as −1 dB for visualization.

There was a statistically significant positive correlation between cone density and visual sensitivity at the locus of fixation (*r*^2^ = 0.4822, *P* = 0.0001) and at 400 µm and 800 µm from the fovea (*r*^2^ = 0.2, *P* < 0.0001, *r*^2^ = 0.13, *P* = 0.0005, respectively). There was no statistically significant relationship seen at 1200 µm, 1600 µm, or 2000 µm (*r*^2^ = 0.02, *P* = 0.3063, *r*^2^ = 0.06, *P* = 0.1839, and *r*^2^ = 0.001, *P* = 0.9211, respectively; Fig. 4).

**Figure 5.**
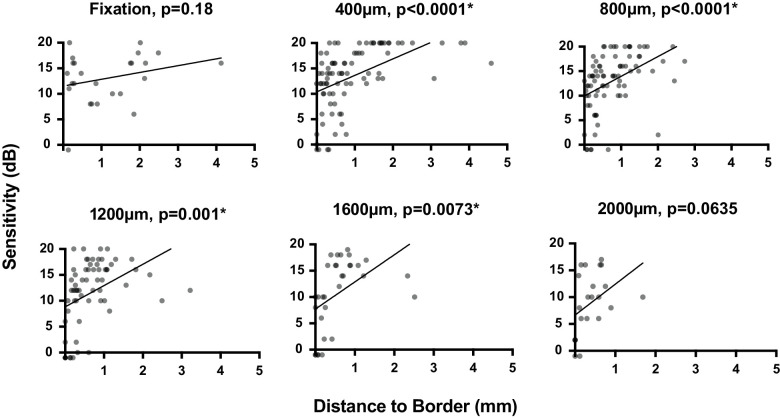
Visual sensitivity compared to distance to the atrophic border across eccentricities. Each panel shows the linear regression comparing microperimetry response with distance to the border at each respective eccentricity. Locations where no sensitivity response was recorded to the brightest visual stimulus are plotted as −1 dB for visualization.

Visual sensitivity was positively related to the distance to the atrophic border at 400 µm, 800 µm, 1200 µm, and 1600 µm (*r*^2^ = 0.26, *P* < 0.0001, *r*^2^ = 0.19, *P* < 0.0001, *r*^2^ = 0.16, *P* = 0.001, and *r*^2^ = 0.22, *P* = 0.0073, respectively). A statistically significant relationship between visual sensitivity and distance to the atrophic border was not seen at fixation (*r*^2^ = 0.07, *P* = 0.18), or at 2000 µm (*r*^2^ = 0.16, *P* = 0.0635), although there was a positive trend at these eccentricities (Fig. 5).

**Figure 6.**
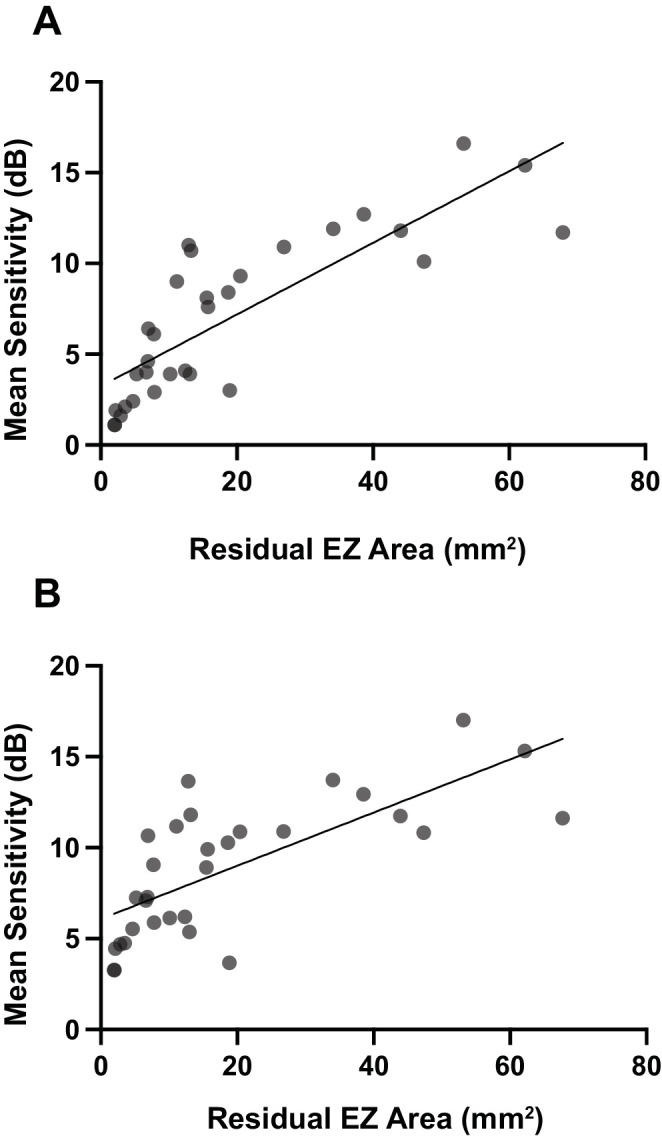
Linear regression comparing mean sensitivity to residual EZ in mm^2^. (**A**) Measurements taken from the device and (**B**) manually calculated mean sensitivities excluding measurements taken from within the region of established atrophy.

The average mean sensitivity from all 41 locations tested with the microperimetry device among the 31 participants was 7 dB with a range of 1.1 to 16.6 dB. When manually calculated excluding regions within the established atrophic zone, this figure was 8.9 dB with a range of 3.3 to 17.1 dB. Five participants had sensitivity measurements > 0 dB out to the full extent of the microperimetry protocol (their retained retinal island extended beyond the testing protocol, therefore no stimuli fell within the region of established atrophy). On average, 28% of microperimetry locations per patient fell outside of the retained retinal island of relative preservation, thus falling within the established region of atrophy. Average EZ area within the retained retinal island was 19.24 mm^2^ with a range of 2.04 mm^2^ to 67.88 m^2^. There was a statistically significant positive relationship between mean sensitivity and EZ area on OCT for both the device reported mean sensitivity (*r*^2^ = 0.68, *P* < 0.0001; Fig. 6A) and the manually calculated mean sensitivity that excluded stimuli falling within the region of established atrophy (*r*^2^ = 0.53, *P* < 0.0001; Fig. 6B).

## Discussion

Our results confirm that sensitivity estimates by microperimetry relate to cone photoreceptor density by AOSLO in CHM. High resolution imaging showed a relationship between the residual cone density and visual sensitivity at discrete points within 1 mm of fixation. This suggests that decreasing photoreceptor densities within the foveal region contribute or drive the decline in central cone vision in CHM and supports the use of AOSLO as a structural outcome measure in clinical trials of therapies for CHM. We also show that visual sensitivity declines with increasing proximity of the atrophic border outside of the center of the fovea, which supports the use of visual sensitivity as an outcome measure at predetermined perifoveal locations sufficiently within the retained retinal area but closer to the atrophic border than the central fovea.

Cone density in CHM is reduced compared with healthy controls at the fovea. We observed a reduction in cone density of up to 50% at fixation (see Fig. 3) in keeping with our previous work examining the peak cone density.^10^ This 50% reduction was despite the relatively normal visual acuity, suggesting that high contrast, high luminance visual acuity is an insensitive marker of central disease severity in CHM. This finding is in agreement with reports from other inherited retinal degenerations in which up to 60% cone reduction could be associated with normal visual acuity.^22^^,^^23^ The association between cone density and mesopic sensitivity estimates within the central retina suggests that further structural loss contributes to continuing functional decline. These findings suggest that strategies to maintain densities and a normal cone mosaic at the foveal center in CHM will maintain visual function and visual acuity. These findings also support the use of structural metrics of the photoreceptor mosaic within the central fovea as meaningful outcome measures.

Although cone densities are significantly reduced at all eccentricities, the densities measured beyond 1 mm from fixation are closer to those in healthy controls (see Fig. 3). At these eccentricities, a correlation between sensitivity and cone density was not found (see Fig. 4). However, we did show a relationship between visual sensitivity and proximity of the atrophic border from 400 to 1600 µm, suggesting that the cone function declines asynchronously to cone density as the region of centripetally advancing retinal atrophy approaches (see Fig. 5). A positive trend between proximity to the border and visual sensitivity also was observed at 2 mm (the widest eccentricity examined), but analysis failed to reach statistical significance at this eccentricity. This may be explained by lower statistical power; only 10 participants had retained retinal structure at 2 mm from the fovea, and therefore the lowest number of ROIs were included at this eccentricity. It is also interesting to note that by definition, these individuals were the participants with the largest retained retinal island and therefore exhibited the least severe foveal/parafoveal disease. At fixation, the region most likely to be furthest from the atrophic border across individuals, a relationship with the distance to the border, was not seen (see Fig. 5), rather foveal sensitivity appears to result from retained cone density (see Fig. 4).

**Figure 7.**
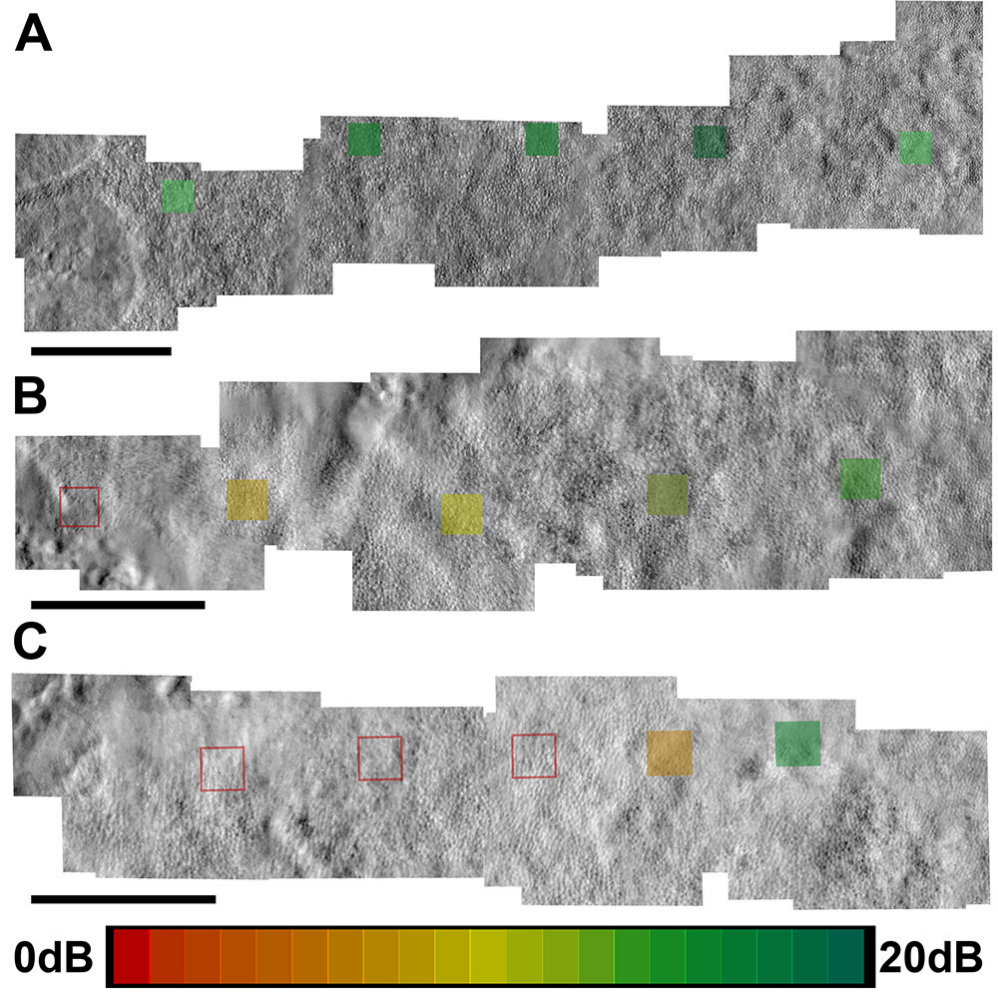
Examples demonstrating the range of sensitivity trends across eccentricity. Split-detection AOSLO montages showing a horizontal (nasal or temporal) arm of three participants demonstrating (**A**) preserved visual sensitivities approaching the atrophic border, (**B**) reducing sensitivity approaching the atrophic border, and (**C**) absent sensitivity proximal to the atrophic border in regions of relatively preserved photoreceptor mosaic. *Colored squares* represent 70 µm × 70 µm ROIs within which cones were identified. The fovea is to the right of the displayed montages for all three participants. *Empty red squares* represent areas in which the brightest visual stimulus was not seen. Corresponding visual sensitivities recorded at each location are indicated by the color of the square corresponding to the color key on the bottom. The 300 µm scale bars are shown for each panel.

There were varying trends in sensitivity change moving away from the foveal center across participants. For example, Figure 7A shows relatively preserved visual sensitivities out to the atrophic border, Figure 7B shows a trend of reducing sensitivity toward the atrophic border, and Figure 7C shows an example of a participant with absent sensitivity (no response to the brightest stimulus) despite an intact cone inner segment mosaic with visible, albeit mottled, outer segment waveguiding at the tested locations. As a result, the maximum potential response to treatment for these patients may be variable, with the patient shown in Figure 7C having the highest potential for functional gains compared to the patient in Figure 7A. Patient enrollment criteria and outcome measure selections for future clinical trials may want to consider these phenotypes in the study design.

We specifically examined ROIs found in a ring within 1.5 mm of the atrophic border to assess for the proportion of retinal regions that might have potential for improvement, excluding ROIs within half a degree of the atrophic border, in which high test-retest variability would be expected.^24^ In our study, 45% of the ROIs contained within this 1.5 mm ring had a sensitivity of ≤ 13 dB, and 24% had a sensitivity of ≤ 13 dB along with a cone density above the median for their eccentricity. The level of ≤ 13 dB was chosen as these ROIs had the potential to improve by 7 dB using the Nidek microperimeter as the testing device.

Previous reports have suggested 7 dB as a clinically significant change in sensitivy.^25^^,^^26^ Using the ROIs examined within the 1.5 mm ring close to but not at the atrophic border, we assessed how many participants in this study had the potential to show a clinically significant improvement in sensitivity, defined as having at least 5 locations with sensitivities ≤ 13 dB. In our study, 29% of participants had 5 or more spots within this ring capable of clinically significant improvement; and 42% of patients had 4 or more spots. Seven of the participants did not have any locations within this ring that were ≤ 13 dB. It is important to remember that our testing strategy only expanded along the four cardinal directions, rather than being spread throughout the full retained island of the central retina. Therefore, there are a limited number of ROIs tested per participant within the 1.5 mm ring. Use of a testing strategy using protocols designed to sample this region more finely for each patient would likely yield a substantial number of points with potential for improvement in most patients. Therefore, we would predict that five or more retinal locations capable of clinically meaningful gains in sensitivity could be prespecified at baseline visits of experimental treatment studies in a high percentage of patients with CHM.

When choosing outcome measures for clinical trials, it is important to consider the method of delivery of the investigational product. Recent clinical trials testing gene augmentation for CHM have involved a surgical procedure to deliver the normal CHM gene to the subretinal space via a subretinal injection.^13^^,^^27^^–^^30^ The mechanical effects of a subretinal injection delivered through an otherwise uncomplicated surgery may structurally damage photoreceptors which may lead to sensitivity losses. The simplest and hardest to prove promise after these genetic treatments is that there will be a stabilization or slowing of the rate of progression post-treatment, although gains in sensitivity may still occur in specific disease stages, retinal locations, or patients. We have documented transient shortening of cone photoreceptor outer segments and lowering of central sensitivities but recovery of both the microscopic cone mosaic as well as the retinal function within 6 months after the interventions,^13^^,^^30^ which was reassuring in terms of safety of the procedure in this specific disease. Sensitivity losses after otherwise uneventful subretinal injections, however, are a well-recognized possibility that may complicate such a desirable outcome by resetting a structural and functional baseline post-intervention against which stability or slowing of progression would need to be measured. This possibility of a negative outcome can certainly create a challenge in demonstrating the efficacy of CHM therapeutics requiring subretinal injections.

Bound cone density was used as the quantitative photoreceptor metric given its stability to the effect of cell identifications at the edge of the ROI.^31^ This characteristic of bound cell density also creates a floor phenomenon, where at least four cells are needed for one to have the possibility of being considered “bound.” The lowest densities generated as part of this study were 1400 cells/mm^2^ with only 11 cells identified in the 70 µm × 70 µm ROI. Nidek-MP1 is capable of detecting sensitivity between 0 dB and 20 dB creating both a floor and ceiling effect on sensitivity measurements. The ability to discriminate lower cone densities and higher sensitivities would likely have strengthened the association relationships described in this study.

Microperimetry is a subjective test with inherent measurement error. Participant inattention may falsely elevate the threshold, resulting in a lower sensitivity measurement. Fixation instability and microsaccades faster than the 25 Hertz (Hz) frame rate of the device may cause microperimetry stimuli to fall outside of the area projected by the device. Mean sensitivity has been advocated as a superior choice of end point in clinical trials due to its higher reliability compared to pointwise assessments.^24^ However, mean sensitivity is a global measure, and given the slow rate of loss of EZ band in CHM,^12^ especially in individuals with advanced disease and minimal residual retinal area, this measure is unlikely to be meaningful in the time course of a clinical trial. Our results support use of regions within the retained retinal island, while using caution in interpretation of results immediately at the atrophic border, where test-retest variability has been reported to be highest.^24^

Longitudinal imaging of individuals with relatively mild disease, and large expanses of retained retina at baseline will help to characterize the rate and pattern of progression of the structural and functional loss. Further studies incorporating cellular scale functional assays such as adaptive optics microperimetry^11^ and optoretinography^14^ to study within and across the retained retinal island could aid in the characterization of structurally present but dysfunctional cells.

## Conclusions

We have demonstrated a structure function relationship within the central retina in CHM. This provides rationale for use of structural outcome measures, such as quantitative metrics of the photoreceptor mosaic on AOSLO and EZ area on OCT, in future clinical trials of therapeutics in CHM. We have also demonstrated a case for use of functional outcome measures, such as microperimetry in the perifovea and approaching the atrophic border.
